# Statistical network analysis for functional MRI: summary networks and group comparisons

**DOI:** 10.3389/fncom.2014.00051

**Published:** 2014-05-06

**Authors:** Cedric E. Ginestet, Arnaud P. Fournel, Andrew Simmons

**Affiliations:** ^1^Department of Mathematics and Statistics, Boston UniversityBoston, MA, USA; ^2^Department of Neuroimaging, Centre for Neuroimaging Sciences, Institute of Psychiatry, King's College LondonLondon, UK; ^3^National Institute of Health Research, Biomedical Research Centre for Mental Health and Biomedical Research Unit for DementiaLondon, UK; ^4^Laboratoire d'Etude des Mécanismes Cognitifs, EA 3082, Université Lyon IILyon, France

**Keywords:** networks, N-back, statistical parametric network (SPN), small-world topology, working memory, weighted density, density-integrated metrics

## Abstract

Comparing networks in neuroscience is hard, because the topological properties of a given network are necessarily dependent on the number of edges in that network. This problem arises in the analysis of both weighted and unweighted networks. The term density is often used in this context, in order to refer to the mean edge weight of a weighted network, or to the number of edges in an unweighted one. Comparing families of networks is therefore statistically difficult because differences in topology are necessarily associated with differences in density. In this review paper, we consider this problem from two different perspectives, which include (i) the construction of summary networks, such as how to compute and visualize the summary network from a sample of network-valued data points; and (ii) how to test for topological differences, when two families of networks also exhibit significant differences in density. In the first instance, we show that the issue of summarizing a family of networks can be conducted by either adopting a mass-univariate approach, which produces a statistical parametric network (SPN). In the second part of this review, we then highlight the inherent problems associated with the comparison of topological functions of families of networks that differ in density. In particular, we show that a wide range of topological summaries, such as global efficiency and network modularity are highly sensitive to differences in density. Moreover, these problems are not restricted to unweighted metrics, as we demonstrate that the same issues remain present when considering the weighted versions of these metrics. We conclude by encouraging caution, when reporting such statistical comparisons, and by emphasizing the importance of constructing summary networks.

## 1. Introduction

Are neurological networks topologically stable across different populations of subjects or across different cognitive and behavioral tasks? This general research program has been carried out by a myriad of researchers in the last decade. Neuroscientists are often interested in evaluating whether the small-world properties of a given brain network are conserved when comparing patients with controls. Bassett et al. (2008), for instance, have studied the differences in anatomical brain networks exhibited by healthy individuals and patients with schizophrenia. Similarly, some authors have tested how the topological properties of certain functional networks are affected by different behavioral tasks (Cecchi et al., 2007; De Vico Fallani et al., 2008; van den Heuvel et al., 2009). Brain network topology has been studied at different spatial scale (Bassett et al., 2006), and different time scales (Pachou et al., 2008; Salvador et al., 2008). It is therefore undeniable that there is considerable academic interest in comparing families of networks; whether these represent several groups of subjects, or the different conditions of an experiment. This general research paradigm is particular amenable to the analysis of subject-specific networks. When such individual networks are available, one can readily compute subject-specific topological measures, which will then be compared across experimental conditions. This type of analysis has been conducted using both functional and structural MRI data (Hagmann et al., 2008; Gong et al., 2009). In this paper, we will mostly focus on networks arising from functional MRI (fMRI) data.

The prospect of performing rigorous statistical analysis of several populations of networks, however, has been hindered by various methodological issues. These statistical questions have not been hitherto satisfactorily resolved in the neuroscience community, and the field of network data analysis remains an area of active methodological development (Simpson et al., 2013a,b). When one is considering the question of comparing several populations of brain networks, two main problems arise. First and foremost, the problem of the inherent dependence between connectivity strength (i.e., wiring density) and network topology (i.e., patterns of edges) necessarily arises. Most, if not all, of the topological metrics that have become popular in the neuroscience literature are highly sensitive to the differences in the number of edges of the graphs under comparison. Therefore, when trying to evaluate the topological properties of different groups of networks on the sole basis of their topology, one also requires to apply some level of control on the differences in density between the groups of networks under scrutiny.

Secondly, the issue of separating differences in density from differences in topology is compounded by the problem of thresholding association matrices. In many cases, neuroscientists are considering correlation matrices with values ranging between −1 and 1. Because network science is founded on graph theory, which is a branch of discrete mathematics, it follows that the application of graph-theoretical methods requires the use of a particular threshold in order to produce adjacency matrices. Naturally, this choice of threshold is often arbitrary, although various statistical strategies have been deployed to alleviate the consequences of such decisions. Several authors have thresholded correlation matrices by applying an inferential cut-off point. This approach is similar in spirit to the standard mass univariate strategy regularly adopted within the context of classical statistical parametric mapping (Friston, 1994).

However, this thresholding of matrices is generally critized for throwing away valuable information. Indeed, since network analysis proceeds by comparing the global topological properties of the graphs obtained after binarizing correlation matrices, it is natural to conclude that a substantial amount of real-valued information has been discarded; and replaced by a sequence of binary digits. As a result, several authors have proposed to use the weighted versions of the classical graph-theoretical measures of topology (Rubinov and Sporns, 2010). It is commonly believed that the use of such weighted topological statistics alleviates both the problem of selecting an arbitrary threshold, and also ensures that one is separating differences in topology from differences in network density. Although this first requirement is indeed satisfied, the second is only illusory. We will show in this paper that the use of weighted topological measures is just as liable to be determined by differences in density, as their standard unweighted versions.

In the present paper, we will concentrate our attention on weighted networks since these are more likely to be found in the biomedical sciences than their unweighted counterparts. This article is structured in two parts. We firstly review how to construct summary networks representing subject-specific or group-specific functional connectivity over time. Here, a mass-univariate approach is adopted using different corrections for multiple comparisons. A similar approach can also be used for representing group differences in functional network topologies. In a second part, we concentrate on network properties inference. This is rendered particularly arduous by the fact that such networks tend to display different number of edges. Since network density is highly predictive of a host of network topological measures, such statistical inference requires special attention, when comparing groups of subjects that exhibit substantial differences in network density.

## 2. Construction of summary networks

We firstly describe how one can construct summary networks from a family of subject-specific weighted or unweighted networks. This task can be tackled by combining the data available, using a mass-univariate approach, as is commonly done in fMRI. Note that the phrases, graph and network, will be used interchangeably in this paper.

### 2.1. Statistical parameter network (SPN)

Here, we review an efficient method for summarizing inference on networks, using a mass-univariate approach. By tacit consensus, this method has essentially become the norm in the field (Achard et al., 2006; He et al., 2007, 2009b; Ginestet et al., 2012). This strategy should be compared to the one adopted in the classical statistical parametric mapping (SPM) framework, which has been utilized in neuroimaging for the past two decades (Friston, 1994). Consequently, this approach will be referred to as statistical parametric networks (SPNs). The problem of constructing a summary graph centers on how to combine the elements of a population of subject-specific correlation matrices. In the SPN framework, summary networks are constructed irrespective of whether or not structural or functional data are being used. While in fMRI studies, it has been common for researchers to compute correlations over time between regions of interest (Achard et al., 2006; Achard and Bullmore, 2007), studies based on structural MRI data, by contrast, have considered between-regions correlations with respect to the available population of subjects (Bassett et al., 2008). In this section, we will concentrate on the specific problem posed by the study of functional MRI cortical networks, where each subject-specific correlation matrix represent inter-regional normalized covariances, computed with respect to a sequence of time points.

Succinctly, one may say that an SPN is to a correlation matrix, what an SPM is to an intensity map. As for the latter, an SPN can be produced in order to obtain a summary network. Different summary networks can be constructed for the different conditions of an experiment, or for the different groups of subjects under scrutiny. Achard et al. (2006) and He et al. (2009b), for instance, have visualized their data using summary networks, whereby an edge is solely included when a corresponding test statistic for that edge is significant. We will refer to such summary networks as *mean* SPNs. Similarly, one can construct *differential* or *difference* SPNs, which represent the edges that have been significantly “lost” and the edges that have been significantly “gained,” when comparing the graphs across experimental conditions, or when considering several groups of subjects. Under its many guises, this approach has been adopted by various authors including Zalesky et al. (2010) and Richiardi et al. (2011), who have used network-based statistics and machine learning methods, respectively, for the comparison of a group of subjects with a group of controls.

The SPN approach that we wish to present here is slightly more general, since it accommodates sophisticated experimental designs, in which information may be pooled over a number of experimental conditions. As for SPM, such analyses enable a concise visualization of the data, which can be interpreted in terms of network properties, topology and community structure. This approach is particularly helpful for an efficient reporting of the experimental results. As mentioned in the introduction, the use of SPNs has the additional advantage of somewhat alleviating the methodological concerns associated with the choice of an arbitrary threshold value; since we are here selecting such cut-off points on the basis of a specific *p*-value. Network thresholding is therefore here supplanted by inference.

**Figure 1:**
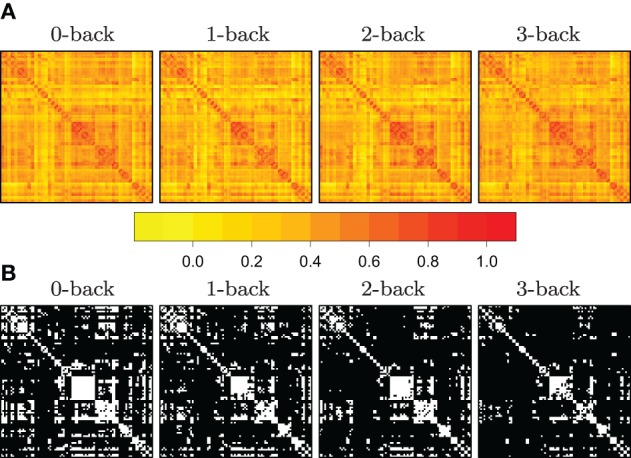
**Illustration of the use of mean SPNs to summarize networks in a cognitive task**. Adjacency matrices become sparser with increasing working memory load. In panel **(A)**, heatmaps corresponding to the correlation matrices in each of four *N*-back conditions, for *n* = 43 subjects. In panel **(B)**, the adjacency matrices were obtained by constructing mean SPNs, using a mass-univariate approach based on *z*-tests with respect to the grand sample mean $\bar{\bar{r}}$ and the grand sample standard deviation sd(**r**) with FDR correction (base rate α_0_ = .05). Zero entries are denoted in black in the adjacency matrices. (See Ginestet and Simmons, 2011 for a full description.)

The thresholding of association matrices, such as correlation matrices, is equivalent to the application of an elementwise indicator function. This type of function, however, is non-linear, in the sense that the sum of the thresholded correlation matrices is not equal to the thresholded mean correlation matrix. That is, this may be formally expressed, as follows,

(1) $$\sum_{i=1}^{n}T_{\tau }(R_{i})\neq T_{\tau }\left(\sum_{i=1}^{n}R_{i}\right),$$

where *i* = 1,…, *n* labels the subjects taking part in the experiment, and where **R**_*i*_'s denote subject-specific correlation matrices. Here, the function, *T*_τ_, is a thresholding function that takes a matrix, and returns its binarized version, with respect to a cut-off point, τ. The issue of thresholding correlation matrices is illustrated in Figure 1, where we have reported some of the preliminary data analysis conducted in Ginestet et al. (2012).

Currently, there is little guidance on how one should proceed, when summarizing the network analysis of a given study. There is hence a pressing need to reach a methodological consensus on how to standardize the construction and reporting of summary networks in neuroscience. A natural desideratum for such summary networks is that they should reflect the topological variability of the entire population of networks. Pioneering work in that direction has been laid out by several authors, including Achard et al. (2006) and He et al. (2009b), for the consideration of a single family of graphs. In the sequel, we review these ideas and extend them to the case of several populations of networks, as was conducted in Ginestet et al. (2012).

The question of drawing inference on families of networks that vary over several experimental conditions can be subdivided into two related issues. On the one hand, one needs to test whether or not the properties of the nodes have been significantly affected by the experimental manipulation. On the other hand, one also needs to evaluate whether or not the presence and absence of edges have significantly varied across the experimental conditions. One can drawn statistical inference for these two distinct, yet related, research questions. Contrary to the classical SPM framework, these two distinct problematics need to be answered using two different types of networks: one for comparing vertices, and another for comparing edges.

A substantial advantage of the SPN methodology is that it addresses the problem arising from the quasi-linearity of the thresholding function presented in Equation (1). Indeed, since we are drawing inference using the correlation coefficients per se, we consequently bypass the problem of averaging over a set of thresholded correlation matrices; while nonetheless producing a statistical summary taking the form of a graph.

We here employ standard graph theoretical notation in order formulate our approach to this specific problem. The interested reader is invited to consult Bollobás (1998) for a more solid introduction to graph objects and their properties. As aforementioned, we will here use the terms networks and graphs interchangeably. In the context of discrete mathematics, a graph *G* is formally defined as an ordered pair of sets (*V*, *E*); in which *V*(*G*) represents the set of *vertices* (sometimes referred to as nodes) in the graph of interest; whereas *E*(*G*) denotes the set of *edges* in that network (also called connections). The total number of edges and total number of nodes in *G* will be concisely denoted by *N*_*E*_ and *N*_*V*_, respectively. A one-way experimental design may be typically composed of *J* experimental conditions, with *n* subjects, per experiment. Thus, the full data set of interest can be described as an (*n* × *J*)-matrix of correlation matrices. In the sequel, the indexes *i* = 1,…, *n* will label the experimental subjects; whereas the indexes *j* = 1,…, *J* will refer to the experimental conditions. Formally, one could represent the full data set as the following matrix,

(2) $$R=\left|\begin{array}{ccc} R_{11} & \ldots & R_{1J}\\ \vdots & \ddots & \vdots \\ R_{n1} & \ldots & R_{nJ} \end{array}\right|.$$

Here, each element **R**_*ij*_ in this equation denotes a correlation matrix of dimension *N*_*V*_ × *N*_*V*_. There is a one-to-one correspondence between each of these correlation matrices and a weighted graphs on *N*_*V*_ vertices or nodes. The individual vertices will be labeled by *v* = 1,…, *N*_*V*_. Moreover, for convenience, each of the matrix entries in **R**, will be denoted by *r*^*e*^_*ij*_; where the superscript *e* labels an edge from the saturated or complete graph, which possesses the maximal number of possible edges. That is, the saturated graph has the following edge set size, *N*_*V*_(*N*_*V*_ − 1)/2. In the rest of this paper, edges will be systematically referred to by using superscripts.

A mean or summary SPN allows to statistically infer the “average” set of inter-regional connections in a group of subjects. Such SPNs are generally obtained by adopting a mass-univariate approach, whereby a sequence of statistical tests are performed for each edge in the edge set. Such an operation may be repeated for each experimental condition. Using the notation introduced earlier, one may conduct a test for each of the columns in the array, denoted **R**, in Equation (2). In effect, we are here considering the following column vectors of correlation matrices,

(3) $$R_{j}={\left[R_{1j},\ldots ,R_{nj}\right]}^{T}.$$

**Figure 2:**
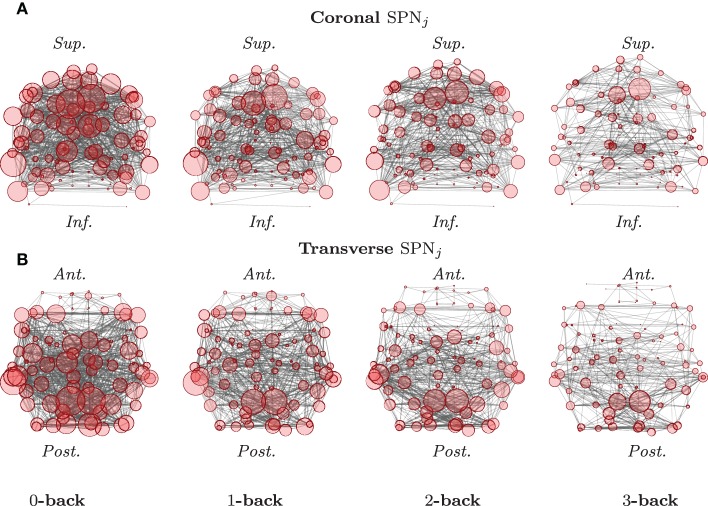
**Graphical representations of mean SPNs over four levels of a cognitive task**. The mean SPNs for an *N*-back task, in the coronal **(A)** and transverse **(B)** planes are here presented, after FDR correction (base rate α_0_ = 0.05). Locations of the nodes correspond to the stereotaxic centroids of the corresponding cortical regions. The orientation axes are indicated in italics: inferior–superior and anterior–posterior for the coronal and transverse sections, respectively. The size of each node is proportional to its degree. (See Ginestet and Simmons, 2011 for a full description.)

Each of these column vectors is analyzed independently in order to produce a single network for each of the different experimental conditions. For the case of correlation matrices, the original matrix entries are routinely Fisher *z*-transformed, in order to be able to use central limit theorems for approximating the density functions of these test statistics. In doing so, one can then draw inference, using an analysis of variance, for instance, or another adequate statistical model, suitable for the data at hand. An example of such mean SPNs under different experimental conditions is reported in Figure 2.

Perhaps, the tenor research question in network data analysis in neuroscience is whether certain edges have been “gained” or “lost,” as a consequence of a particular experimental condition. This general research question can be specifically answered by computing two distinct differential networks, representing what we may call the *downweighted* and *upweighted* SPNs. These two types of networks will be denoted by SPN_−_ and SPN_+_, respectively.

As for mean SPNs, the construction of these differential networks can similarly be conducted within a mass-univariate approach. For differential SPNs, however, statistical inference needs to be drawn from the full data set. That is, one needs to consider all the correlation coefficients described in Equation (2) –that is, the elements contained in the matrix **R**. Computing a differential SPN will generally involve *N*_*E*_ linear models. Depending on the general experimental framework adopted by the researchers, these linear models could be extended to mixed effects models. In its most general formulation, we may consider a repeated block design, which can be succinctly expressed by using the classical formalism due to Laird and Ware (1982),

(4) $$r_{i}^{e}=X_{i}^{e}\beta^{e}+Z_{i}^{e}b_{i}^{e}+\epsilon_{i}^{e};\;i=1,\ldots ,n.$$

Here, each vector, **r**^*e*^_*i*_ = [*r*^*e*^_*i*1_,…, *r*^*e*^_*iJ*_]^*T*^, denotes the correlation coefficients of interest, and β^*e*^ = [β^*e*^_1_,…, β^*e*^_*J*_]^*T*^ consists of the vector of fixed effects. The latter does not vary over subjects and will be the main object of study. By contrast, the **b**^*e*^_*i*_'s are the vector of subject-specific random effects, which will be integrated over. Finally, ϵ^*e*^_*i*_ = [ϵ^*e*^_*i*1_,…, ϵ^*e*^_*iJ*_]^*T*^ is the vector of residuals. Crucially, the **X**_*i*_'s and **Z**_*i*_'s denote the design matrices for the fixed and random effects, respectively. As in standard applications of mixed effects models, the covariance matrices for ϵ^*e*^ and **b**^*e*^ can be assumed to be diagonal and positive semi-definite, respectively (see Demidenko, 2004, for details).

In general, one may include an edge in a differential SPN, when the corresponding *F*-test for the experimental factor has been found to be significant. Depending on the linear model used, different statistical test may be performed (Pinheiro and Bates, 2000). Therefore, the use of a mass-univariate approach for extracting between-condition differences in the presence or absence of edges, yields two different types of differential SPNs. That is, depending on the sign of the significant fixed effect coefficients, one may include that edge in either a downweighted network, which may be denoted SPN_−_; or in an upweighted network, denoted SPN_+_.

A similar approach can be adopted to estimate the upweighting and downweighting of the signal of interest at single *nodes*. Again, such a node-specific differential SPN can be obtained by performing a set of *N*_*V*_ linear models. In this case, the data under consideration is the set of matrices **Y**^*v*^ = {*y*^*v*^_*ij*_}, where each *v* ∈ *V* is a region of interest. Every *y*^*v*^_*ij*_ corresponds to a time-averaged intensity signal, for the *v*th region, for subject *i*, under the *j*th experimental condition. Thus, one could reformulate the system of equations for evaluating edges in (4) by using superscripts to denote vertices.

**Figure 3:**
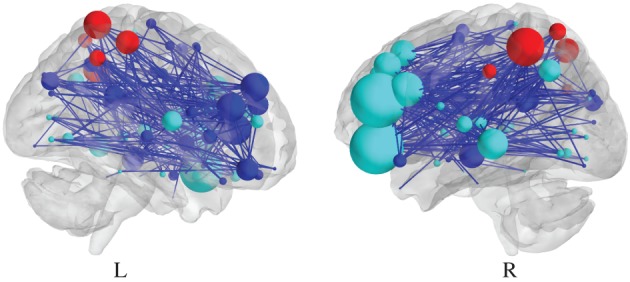
**Visualization of a differential SPN, summarizing the effect of a cognitive experimental factor**. Sagittal section of a negative differential SPN, which represents the significantly “lost” edges, due to the *N*-back experimental factor. The presence of an edge is determined by the thresholding of *p*-values at 0.01, uncorrected (see Ginestet and Simmons, 2011, for a description of the data at hand.).

As for edge-specific differential SPNs, a vertex would be estimated to be either significantly upweighted or downweighted, depending on the sign of the largest coefficient in the corresponding vector β^*v*^. An illustration of such a differential SPN, based on the *N*-back data set, analyzed by Ginestet et al. (2012) is reported in Figure 3. Naturally, this assignment based on the sign of the fixed effects is only possible, when the task under scrutiny is based on an experimental gradient. An alternative strategy may be required, when different levels of the task are expected to affect the response in different directions.

A singular limitation, however, affects all mass-univariate approaches. Such a repetitive use of classical inferential threshold, may lead to a corresponding increase in Type I error. This issue can be addressed by correcting for multiple comparisons. The significance of edges and nodes in both mean and differential SPNs can, for instance, be inferred using the false discovery rate (FDR) with a base rate of α_0_ = .05 (Benjamini and Hochberg, 1995; Nichols and Hayasaka, 2003). Naturally, other corrections for multiple comparisons could also be utilized (see Meskaldji et al., 2011, for a different approach). The conventional thresholding method used in network analysis is therefore superseded by the application of standard multiple testing corrections. The main advantage of this approach lies in its pooling of information over several subjects, in order to produce robust edge- and node-specific statistics.

## 3. Comparison of functions on networks

We now turn to the issue of comparing various types of topological measures over several families of networks (van Wijk et al., 2010). Inference on quantities such as characteristic path length, clustering coefficient and modularity structure has attracted a sustained amount of interest in the neuroscience community. Comparisons of this type of topological measures, however, is generally regarded to be hard, since these topological differences highly depend, in a non-linear fashion, on group differences in edge density.

### 3.1. Global efficiency

One of the classical exemplars of a topological summary of a network is its characteristic path length. Such a quantity, however, is solely defined for connected graphs. The global efficiency of a graph, by contrast, can be computed for any network –connected or disconnected– and is inversely related to its characteristic path length. Efficiency is formally defined by the following formula due to Latora and Marchiori (2001),

(5) $$E(G)=\frac{1}{N_{V}(N_{V}-1)}\sum_{i\in V}\sum_{j\neq i\in V}d_{ij}^{-1},$$

with *N*_*V*_ = |*V*|, as before. Here, *d*_*ij*_ denotes the length of the shortest path between vertices *i* and *j*. Moreover, the second summation is performed with respect to the set, {*j* ≠ *i* ∈ *V*}, which is the set of all indices in *V* that are different from *i*. This efficiency measure can be shown to be equivalent to the inverse of the harmonic mean of the length of the shortest paths between each pair of nodes in the network *G*.

Specifically, the quantity in Equation (5), is usually referred to as the global efficiency of a particular graph, and is denoted by *E*Glo(*G*) = *E*(*G*). Intuitively, this quantity can be understood as the amount of potential information transfer that can be performed in parallel. A local measure of efficiency can also be computed, which is equivalent to the clustering coefficient. For a review of other efficiency measures that have been studied in the context of neuroscience, the reader is referred to Ginestet et al. (2011). The most commonly adopted approach to network comparison is therefore to compute a topological metric, such as global efficiency, for each individual subject, and thereafter to evaluate whether this measure differs over the different experimental groups under scrutiny.

### 3.2. Density-integrated measures

An alternative approach to the problem of quantifying the topology of weighted networks proceeds by integrating the metric of interest with respect to different density levels. Different approaches have been adopted in practice. While some authors have integrated over a subset of the density range (see Achard and Bullmore, 2007, for example), others have integrated over the entire range of densities (He et al., 2009a). The family of topological measures, which is obtained after integrating over different density levels, will be referred to as density-integrated measures. Given a weighted graph *G* =(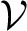, 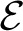, 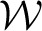), the density-integrated version of the efficiency in Equation (5) can, for instance, be defined as follows,

(6) $$E_{K}(G)=\int E\left(\gamma (G,k)\right)p(k)dk,$$

where density is treated as a discrete random variable *K*, with realizations in lower case, and *p*(*k*) denotes the probability density function of *K*. Since *K* is discrete, it can only take a countably finite number of values. In general, it is common to assign equal weight to every possible choice of density.

The function γ(*G*, *k*) in Equation (6) is a density-thresholding function, which takes a weighted undirected network and a level of wiring density as arguments, and returns an *unweighted* network. Since there is no prior knowledge about which values of *K* should be favored, one can specify a uniform distribution on the set of all possible densities. Note, however, that other distributions could be selected for this purpose (see Ginestet et al., 2011, for a discussion of alternative specifications).

### 3.3. Integrating over densities

The question of separating topology from density could be reformulated as the statistical problem of evaluating topological differences, while “controlling” for differences in density. When adopting this perspective, it is convenient to treat topology and density as random variables. We have already done so for density, in the previous section. Implicitly, by integrating over all possible thresholds, we are indeed considering density as a random variable with a well-defined probability distribution, which is, in the present case, a uniform distribution.

A natural desideratum, which may be required when comparing network topological characteristics, while controlling for differences in topology; would be to control for weighted networks whose association matrices are proportional to each other. That is, if two different matrices are linearly related to each other, it seems reasonable to conclude that their topologies must be identical, after one has controlled for such a linear difference in density. Thus, consider the following simple example, adapted from Ginestet et al. (2011).

**Example 1** (Ginestet et al., 2011). We here have two networks, *G*_1_ and *G*_2_, with proportional association matrices **W**_1_ and **W**_2_, satisfying **W**_1_ = α**W**_2_. That is, these two matrices are proportional to each other. An application of the density-integrated metrics described in Equation (6) to these networks would give the following equalities,

(7) $$E_{K}\left(W_{1}\right)=E_{K}\left(\alpha W_{2}\right)=E_{K}\left(W_{2}\right).$$

That is, when integrating with respect to density, we are in fact evaluating the efficiencies of *G*_1_ and *G*_2_ at a number of cut-off points. At each of these points, the efficiency of the two networks will be identical, because **W**_1_ is proportional to **W**_2_ and therefore the same sets of edges will be selected. Therefore, *G*_1_ and *G*_2_ have identical density-integrated efficiencies.

While illustrative, this example is not entirely satisfying. In fact, this result can be shown to hold in a more general sense. The invariance of density-integrated efficiency turns out to be true for any monotonic (increasing or decreasing) function *h*, as formally stated in the following result.

**Proposition 1** (Ginestet et al., 2011). *Let a weighted undirected graph G* = (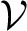, 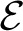, 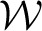). *For any monotonic function h*(·) *acting elementwise on a real-valued matrix* **W**, *and any topological metric E, the density-integrated version of that metric, denoted E_K_, satisfies* 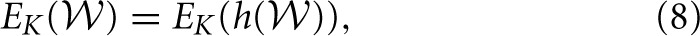 *where we have used the weight set*, 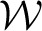, *as a proxy for graph G*.

A proof of this proposition can be found in Ginestet et al. (2011). The demonstration essentially relies on the fact that any monotonic transformation of the entries of a real-valued matrix will preserve the ranks. Therefore, proposition 1 makes rigorous a potential way of “controlling” for differences in density. That is, this formal proposition states that we are indeed controlling for any monotonic transformation of the original entries in the matrix. In effect, proposition 1 should be regarded as a potential definition of what it means for two networks to solely differ in terms of topology, while controlling for monotonic differences in density.

### 3.4. Density and modularity

Another network property, which has been studied extensively in the literature is modularity structure. As for efficiency and other topological measures, however, modularity is also highly dependent on edge density. Therefore, any attempt at comparing the modularity of different groups of networks will be confounded by group differences in the networks' number of edges. We illustrate this problem with the results reported in a recent paper by Bassett et al. (2011), who have analyzed the static and dynamic organization of functional brain networks in humans. We here focus on the first claim made in this paper, which states that the static modular structure of such networks is nested with respect to time. In particular, Bassett et al. (2011) argue that this graded structure underlines a “multiscale modular structure.”

As for global efficiency in the previous section, it can be shown that modularity structure is substantially mediated by edge density. In the case of weighted networks, this is equivalent to a difference in the size of the correlation coefficients. In Bassett et al. (2011), for instance, the authors report that the size of the mean correlation diminishes with the size of the time window. Such a decrease in overall correlation will generally have two effects: (i) networks' topologies will become increasingly more “random” and (ii) the number of significant edges will decrease. Here, we use synthetic data sets to show that these two phenomena are likely to be associated with a higher number of modules, thereby potentially explaining the apparent multiscale modular structure described by Bassett et al. (2011). Our simulations are based on the unweighted unsigned version of the modularity algorithm of Clauset et al. (2004), but may be extrapolated to weighted signed adjacency matrices.

**Figure 4:**
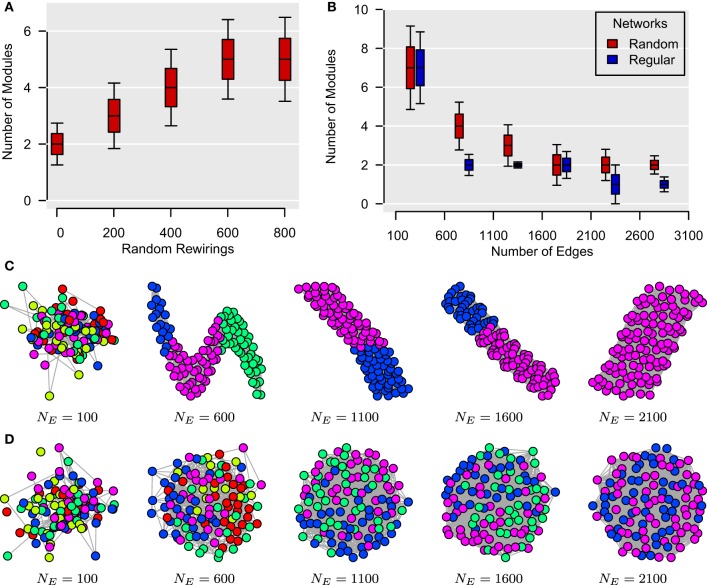
**Topological randomness and number of edges predict number of modules. (A)** Relationship between the number of random rewirings of a regular lattice and the number of modules in such a network. Here, the number of edges is kept constant throughout all rewirings. **(B)** Relationship between the number of edges in a network and its number of modules for both regular (i.e., lattice) and random graphs. This shows that the number of modules tends to decrease as more edges are added to both types of networks. **(C,D)** Modular structures of regular **(C)** and random **(D)** networks for different number of edges, *N*_*E*_. These networks are represented using the algorithm of Kamada and Kawai (1989) with different colors representing different modules. In all simulations, the number of vertices is *N*_*V*_ = 112, as in Bassett et al. (2011).

In Figure 4A, we have generated 1000 unweighted lattices based on 112 vertices as in Bassett et al. (2011). By randomly rewiring the edges of these lattices, we show that the number of modules in these networks tends to increase with the level of topological randomness in these graphs. For Figures 4B–D, we have generated two sets of unweighted networks, characterized by a random and a regular topology, respectively, with different number of edges. These simulations were repeated 1000 times for each type of graph for each number of edges. For both types of networks, the number of modules in these graphs tended to decrease as new edges were added. Collectively, although these data simulations do not entirely rule out the possibility of a temporally nested modular structure in the human brain, they nonetheless cast doubts on the possibility of detecting such a temporal organization by reducing the size of the sampling window. Such subtle artifactual relationships between modularity and edge density can arise in a range of different settings in the analysis of neuroimaging data.

### 3.5. Weighted topological metrics

Since the previous two sections have highlighted the potential problems associated with thresholding correlation matrices, one may surmise that such problems could be adequately dealt with, by directly considering the weighted versions of the topological metrics of interest. In particular, an apparently natural way of combining differences in density with differences in topology is to consider the weighted versions of traditional topological metrics. For the aforementioned global efficiency, for instance, one can define a weighted global efficiency, denoted *E*_*W*_, as follows, 
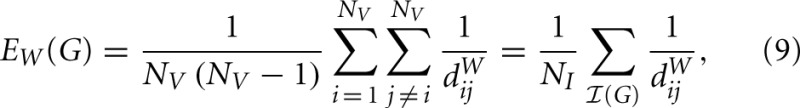
 where *d*^*W*^_*ij*_ represents the weighted shortest path between the *i*th and *j*th nodes. Unfortunately, another theoretical result points to a serious limitation of *E*_*W*_, which may potentially dissuade researchers from using this particular type of metrics. With the next proposition, we demonstrate that under mild conditions, the weighted efficiency is simply equivalent to the weighted density, sometimes referred to as weighted cost, of the graph of interest,

(10) $$K_{W}(G)=\frac{1}{N_{V}\left(N_{V}-1\right)}\sum_{i=1}^{N_{V}}\sum_{j\neq i}^{N_{V}}w_{ij}.$$

**Proposition 2** (Ginestet et al., 2011). *For any weighted graph G* = (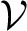, 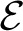, 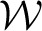), *whose weighted edge set is denoted by* 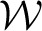
(*G*) = {*w*_*ij*_: *i* < *j*}, *if* 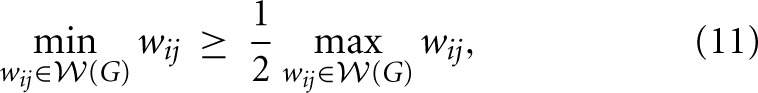 then

(12) $$E_{W}(G)\;=\;K_{W}(G).$$

A proof of this result can be found in Ginestet et al. (2011). Not surprisingly, proposition 2 places emphasis on the spread of the distribution of the weighted edge set 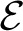
(*G*). The condition in proposition 2 may at first appear quite constraining. However, this condition encompasses a wide range of experimental situations, including the data set described in Ginestet and Simmons (2011). Thus, the added benefit of utilizing the weighted version of the global efficiency measure may, in most settings, be highly questionable, since there exists a one-to-one relationship between this topological measure and a simple average of the edge weights. Cutoff-integrated efficiency and other cutoff-integrated measures, as described in Ginestet et al. (2011), may therefore be preferred, in practice, when one wishes to summarize the influence of both density and topological differences.

## 4. Conclusion

In this paper, we have briefly reviewed some of the methodological research that has been conducted on network data analysis, as applied to functional neuroimaging. Two main threads ran through this discussion. Firstly, we considered the different approaches that one may adopt, when summarizing several subject-specific networks. Secondly, the thorny issue of graph thresholding was also tackled, with special emphasis on the comparison of network modularity and the use of weighted topological metrics.

From the above discussion, it should be clear that there does not exist a single way of computing a mean network. This is, in some sense, an ill-defined problem. A commonly adopted perspective on this issue is to perform a mass-univariate test, where the significance levels of every edge are evaluated, and then thresholded. We have seen that this approach can be carried out both within a single family of networks, and over an entire experimental design, using a mixed effects model. By analogy with the classical SPM approach used in neuroimaging, one may refer to such uses of a mass-univariate approach on networks, as SPNs.

Secondly, we have discussed one of the long-standing issues in the application of network data analysis to neuroscience data: the question of whether or not one should threshold matrices of correlation coefficients, for the purpose of producing adjacency matrices. In this paper, we have reviewed a range of different approaches to this problem. On the basis of the several examples and counterexamples that we have studied, we are able to make a few methodological recommendations to researchers in the neuroscience community, intending to compare the topological properties of two or more populations of weighted networks. Note that these recommendations are solely tentative, as no general consensus has yet been reached on this particular issue.

As a first step, we argue that it is good practice to standardize the association weights. This may facilitate comparison across distinct network analyses, and ease the interpretation of the results. Secondly, the weighted density, or connectivity strength, of the networks of interest should then be reported. This is central to the rest of the analysis, and therefore, this quantity should be computed and reported systematically. Indeed, if the groups of networks under scrutiny substantially differ in terms of average density, then these differences are highly likely to affect any comparison of the topological properties of these groups of networks. Finally, population differences in density-integrated topological metrics may then be evaluated and reported. This will indicate whether the topologies of the populations under scrutiny vary significantly after having controlled for monotonic differences in connectivity strength.

The theoretical results described in this paper have only been presented for the global efficiency metric. Thus, these propositions and the examples studied need not necessarily apply to other topological measures. However, we also note that proposition 1 has been proved with a high degree of generality. This proposition and its proof is indeed independent of the particular formula of the metric of interest, and therefore could easily be extended to any other function of the weighted graph matrix. In particular, because most weighted metrics are constructed on the basis of the matrix of weighted shortest paths, one surmises that this theoretical result may, in fact, hold in a more general setting.

Importantly, we have also shown that network modularity is not immune to this dependency on edge density. If several populations of networks differ in their number of edges, then it is likely that the resulting group-specific modularity structures will not be comparable. That is, such comparisons will mainly reflect differences in edge density, and as such may not carry much explanatory power. This is an area of application of statical network analysis, where one should exert caution, as the powerful algorithms used for detecting network modules may hide the potential confounding effects of differences in edge density.

Finally, the use of weighted topological metrics was also considered. Unfortunately, we have seen that simply replacing classical network measures by their weighted analogs is not sufficient to resolve the dependency of these measures on edge density. Thus, cutoff-integrated topological measures, such as the cutoff-integrated efficiency described in Ginestet et al. (2011), may be preferred in practice, when one wishes to separate differences in edge density from differences in topology.

## Author contributions

Cedric E. Ginestet has written the review. Andrew Simmons has provided data and has reviewed the paper, whereas Arnaud P. Fournel has produced some of the figures. Arnaud P. Fournel has also assisted with the revisions of the paper.

## Conflict of interest statement

The authors declare that the research was conducted in the absence of any commercial or financial relationships that could be construed as a potential conflict of interest.
