# Supplementary material for: Statistical network analysis for functional MRI: summary networks and group comparisons
Source: Front Comput Neurosci. 2014 May 6;8:51. doi: 10.3389/fncom.2014.00051 (PMC4018548; doi:10.3389/fncom.2014.00051)
Supplement: Supplementary file 1 [file Presentation1.PDF]

Replies to Reviewers

Cedric E. Ginestet, Arnaud Fournel, and Andrew Simmons

Statistical Network Analysis for Functional MRI:
Mean Networks and Group Comparisons.

We thank the two reviewers for their efforts and patience in reading this manuscript. We have addressed the main concerns of the reviewers, which appear to center on two points:

i. The manuscript lacked a clear structure. It was halfway between a review paper and a primary data paper. We have tried to re-center and simplify the presentation of the manuscript, in order to make its format closer to the one of a review paper.

ii. Both reviewers have also pointed out that some mathematical notation was unnecessary and could be simplified. We have also attended to these comments by suppressing some redundant notation, equations, figures and examples.

1 Reviewer 1

This paper describes various statistical approaches for comparing brain networks. Statistical comparison of the connectome is a timely topic, as evidenced by its prominent coverage in the recent special image of NeuroImage. Many of the approaches presented in this paper have been demonstrated in the authors earlier work. The paper is presented well, but contains a great deal of mathematical notation, some of which may be considered superfluous. Perhaps consideration can be given to omitting superfluous notation with hope of improving paper readability. Examples are provided below. The power of the statistical test described on page 6 seems to be inferior to a one-way ANOVA for the example given (see below). Consideration should be given to providing an example where the Frechet based t-statistic outperforms a univariate approach in terms of power.

SPECIFIC REMARKS:

1. Statistic on page 6: Consider the case of only two groups. In this case, equation (6) gives that $z_1^e = z_2^e$ for all e. This indicates that either both groups are significantly different than the grand mean (alternative hypothesis) or both groups are equal to the grand mean (null hypothesis). It is not clear why the group mean being equal to the grand mean is a relevant null hypothesis. Note that this is not equivalent to the null hypothesis tested with an ANOVA or t-test, which is that the group 1 mean is equal to the group 2 mean. The ANOVA and t-test provide greater power to detect between-group difference in means compared to the statistic proposed on page 6. This can be trivially tested for the case of two groups. It needs to be demonstrated why the proposed test statistic should be used in favor of an ANOVA.

We thank the reviewer for this very useful observation. The reviewer is entirely right, and we apologize for such a stupid mistake. Indeed, this test is not equivalent to a classical ANOVA. Note that our problem was solely in describing the test, and not in its implementation, since we have used standard routines available in the R language.

Because of this mistake, and in an effort to cut unnecessary mathematical notation, we have thus suppressed that paragraph, and simply replaced it by the following sentence: *When these matrices are correlation matrices, these coefficients are usually Fisher z-transformed in order to permit the application of normal theory for the distribution of these quantities. It is then possible to draw inference, using a standard analysis of variance.*

The use of ANOVA in this context is justified by our preliminary use of the Fisher's z-transform on every correlation coefficient in the sample. The mean networks will be denoted by $\overline{\text{SPN}}_j$ *for each of the* $j = 1, \ldots, J$ *conditions. An example of the application of this type of mean SPN analysis is reported in figure 2, for an experimental data set described in Ginestet and Simmons (2011), under four different N-back conditions.*

2. It is not clear how the SPN framework fits in with network based statistics and the adaptive approach recently proposed by Meskaldji et al. Is it possible for the NBS to complement the the SPN framework to provide a more powerful approach to control for multiple comparisons across all edges than the currently suggested FDR methods? Why is FDR the only approach the can be used with SPN?

The FDR is not the sole thresholding approach that can be used in this context. In order to reflect the great diversity of strategies that can be implemented in this context, and to indicate these recent developments, we have modified this part of the manuscript, and added a reference to the work of Meskaldji et al. (2011). *Finally, as with all mass-univariate approaches, one needs to correct for multiple comparisons. The significance of edges and nodes in both* $\overline{\text{SPN}}_j$ *and* $\text{SPN}_{\pm}$ *can be inferred using the false discovery rate (FDR) with a base rate of* $\alpha_0 = .05$ *(Benjamini and Hochberg, 1995, Nichols and Hayasaka, 2003). Naturally, other corrections for multiple comparisons could also be utilized, such as the recently introduced network based statistics (NBS) approach, due to Meskaldji et al. (2011).*

3. The paper contains a great deal of mathematical notation, some of which may be unnecessary. Some examples: Introducing δ_0 at the bottom of page 9 is probably superfluous since $\delta_0 = 0$ for the vast majority of practical hypothesis. I cant think of an example mandating nonzero δ_0. Introducing p(k) in (16) is probably superfluous. I cant think of a scenario where one would not use p(k)=1/N for all k (i.e. uniformly distributed). Notation and definition of a graph on page 5 is perhaps too technical for a neuroscience journal.

We agree with the reviewer that this paper can be substantially simplified. We have removed the use of δ_0, when describing the Frechet mean framework. This now gives: *The null hypothesis stating that the (restricted) Frechet means of these two distributions are not separated, corresponds to* $H_0 : d(\mu_1, \mu_2) = 0$.

In addition, throughout the paper, we have eliminated the use of := and =: to indicate definitions and replaced this notation by a simple equal sign. Moreover, as suggested by the reviewer, we have also amended the mathematical definition of a graph, by diminishing the amount of formalism: *We employ standard graph theoretical notation to introduce our approach to the above problem (Bollobás, 1998). A graph G is formally defined as an ordered pair* (V, E), *where* $V(G)$ *is the set of vertices (or nodes) in the graph and* $E(G)$ *is the set of edges (or connections). The total number of edges and total number of nodes in G will be denoted by* N_E *and* N_V, *respectively.*

Another paragraph has been removed from the paper, in section 3.2, as that section seemed to provide too many technical details. Finally, a simple example at the beginning of section 3.3 has also been deleted in the revised manuscript.

4. Frechet Mean Network: Interesting approach. The metric d requires definition. A disadvantage of this approach is the lack of localizing power, in the sense

that the null hypothesis cannot be rejected at the resolution of individual edges. Is it possible to compare the power (sensitivity/specificity) of this approach with typical approaches that perform inference at the edge level (e.g. FDR)? With edge level approaches it could be assumed that the network-wide hypothesis is rejected if one or more edge hypotheses are rejected. Simulated data could be used for this experiment.

We thank the reviewer for these particularly useful observations. A range of different metrics can be used in order to measure distances between graphs. We have cited the Hamming distance, as an example, in the revised manuscript. The suggestions made by the reviewer are all very interesting, but would probably deserve to be treated in a separate publication. 'Global procedures' based on distances between Frechet means are mainly appealing because they are applicable irrespective of standard normality assumptions. However, for this reason, they are unlikely to match the power of classical statistical methods, when such assumptions are satisfied. The possibility of comparing edge-level tests with graph-level tests is also intriguing. This is not something that we have yet considered in our research. However, it is unclear how one could select a criterion that would permit to compare these two families of tests: Shall we examine their discriminatory powers at the edge level or at the graph level? We will most certainly try to explore these avenues in future work.

5. Proposition 1 might be considered trivial. It more or less asserts that a rank ordering is preserved under a monotone remapping. Also, the significance/relevance of this proposition is unclear, given that it is very unlikely that the relation between a group of connectivity matrices derived from different individuals can be expressed a monotone relation.

The reviewer is right to point out that both the statement and the proof of this particular proposition are relatively straightforward. However, this proposition is important, because it encapsulates what we mean, when controlling for differences in density. The following sentence has been added in order to emphasize that specific point: *In effect, proposition 1 should be regarded as a potential definition of what it means for two networks to solely differ in terms of topology, while controlling for monotonic differences in density.*

6. Proposition 2: Does condition (28) typically hold in practice? It would seem min(w_i, j) would be close to -1, given the finite (relatively small) number of samples in an fmri time series. This can be trivially assessed, perhaps using some of the publicly available connectome maps or fmri data.

We thank the reviewer for his/her observation. We agree with the reviewer that the condition of proposition 2 is likely to be satisfied in practice. However, testing this condition on a range of publicly available data sets is outside the scope of this review paper.

TRIVIAL

- P 6: Inference testing is here directional.. How can it be directional if the abs is taken in (6)?

As we have mentioned before, this entire paragraph reflected some substantial erroneous thinking on our part. We have eliminated that paragraph and simplified the presentation of this part of the paper. The faulty sentence is not present in the revised manuscript.

- Typo in (7). No need to negate the z-stat? - Relevance of Figure (4) is unclear. What measure are the three simulated scenarios evaluating? Further details are required.

As above, this sentence is not present in the revised manuscript. Moreover, as suggested by the reviewer, this section of the paper has also been simplified. We have entirely removed Figure 4, and have also eliminated the paragraph describing it. Hence, we are left with a more self-contained description of the Frechet mean statistical strategy. Note that relevant references have been left unscathed, so that the interested reader retains the opportunity of exploring this aspect of the paper further.

SUMMARY and RECOMMENDATIONS:

Well-presented paper that addresses the very timely topic of statistical comparison on brain networks (connectome). Readability can be improved by omitting superfluous nomenclature. Consideration should be given to justifying the use of the null hypothesis assessed by the statistic described on page 6; in particular, group mean = grand mean versus the more typical group mean 1 = group mean 2. Power evaluation of the Frechet statistic would add value to this work. Adaptive statistics and the NBS seem to complement the SPN framework and provide an alternative to FDR (i.e. SPN + adaptive/NBS/FDR, but not SPN or adapative/NBS/FDR. Clarification on this point may be required.

We have addressed all these points above in our replies.

REFERENCES: Adaptive strategy for the statistical analysis of connectomes, Djalel Eddine Meskaldji, Marie-Christine Ottet, Leila Cammoun, Patric Hagmann, Reto Meuli, Stephan Eliez, Jean Philippe Thiran, Stephan Morgenthaler, Plos One 6 (2011), no. 8, e2300.

2 Reviewer 2

Overall, the paper stands to contribute to the current literature on network methods and addresses important topics within this area. In what follows, I provide several remarks that I feel would help strengthen the paper.

Comments:

1. The paper seems to begin as a review paper, but then parts of the paper (primarily in sections 2 and 3) seem to shift toward one making original methodological contributions. It would be helpful for the paper to follow a consistent style. If it is a review paper, it would be helpful for the authors to further synthesize the work that is presented and make comparisons between (or discuss strengths and weaknesses of) competing methods. If it is not a review paper, the paper would be enhanced by the inclusion of data examples, empirical demonstrations of operating characteristics of some of the methods, empirical comparisons with competing methods (when applicable), etc.

We thank the reviewer for his/her suggestion. Efforts have been made to make this manuscript closer to a review paper. In particular, we have re-written parts of section 2, to ensure that this indicates other perspectives on the construction of network summaries. Note, however, that the mass-univariate approach has already been de facto accepted as the norm in the field. We cite several references that attest of this wide level of acceptance.

In section 3, by contrast, we are reviewing some technical results that pertain to the comparison of functions on networks. Unfortunately, there are few other papers that have addressed this issue formally, except for an influential paper by van Wijk et al. (2010), which we have now added in

the revision manuscript. In addition and throughout the paper, we have also emphasized the use of the term "review", as in the following passage in the introduction: *This paper is structured in two parts. We firstly review how to construct summary networks representing subject-specific or group-specific functional connectivity over time.*

Also, we have modified the beginning of section 2.1., such that: *We here review an efficient method for summarizing inference on networks, using a mass-univariate approach, which has become the norm in the field (Achard et al., 2006, Ginestet et al., 2012, He et al., 2007, 2009). This strategy is similar in spirit to the one adopted in the statistical parametric mapping (SPM) framework (Friston, 1994), and will therefore be referred to as a statistical parametric network (SPN).*

2. P. 4. Extensive parallels are drawn between SPN and SPM (especially in paragraph two) before SPN is even described. After brief mention of SPM in the first 2-3 sentences of subsection 2.1, it would be helpful to present SPNs and then provide interpretive remarks, which can include parallels draw to SPM.

We agree with the reviewer that this aspect of the manuscript was not very well-structured. Consequently, we have removed some redundant historical remarks about SPM, and re-focused the beginning of that section on SPN. This gives the following revised paragraph: *We here review an efficient method for summarizing inference on networks, using a mass-univariate approach, which has become the norm in the field (Achard et al., 2006, Ginestet et al., 2012, He et al., 2007, 2009). This strategy is similar in spirit to the one adopted in the statistical parametric mapping (SPM) framework (Friston, 1994), and will therefore be referred to as a statistical parametric network (SPN). In recent years, network data analysis in neuroscience has adopted a similar approach by combining networks over a population of subject-specific correlation matrices. In this framework, networks constructed on the basis of structural and functional data are treated in a different manner. Whereas in fMRI studies, researchers have generally computed inter-regional correlations over time (Achard et al., 2006, Achard and Bullmore, 2007), studies based on structural MRI data have utilized inter-regional correlations over subjects (e.g. Bassett et al., 2008). We will concentrate on the specific problem posed by the study of functional MRI cortical networks, where correlation matrices are subject-specific with regional covariances computed with respect to time.*

3. P. 4, first sentence of paragraph two: clarify intensity map. This could refer to many things, e.g. subject-specific maps in a given scan, group-level estimates pertaining to an experimental task (beta map), etc.

Our objective in this paragraph was to convey the idea that SPNs and SPMs are analogous to each other. While it is true that SPMs can be applied to a wide range of different intensity maps, we do not believe that this detracts the reader from the main analogy that we want to build in that sentence.

4. P. 4, paragraph two: SPNs lead to the choice of a different threshold, the pvalue, but does not avoid the use of a threshold altogether. Clarify.

We absolutely agree with the reviewer, and we thank him/her for making that observation. Again, our perspective in this paragraph was to refer to the fact that the arbitrariness of the choice of threshold is alleviated by the use of a formal statistical framework. However, we certainly agree with the reviewer that this does not dispense us from any form of thresholding. The following sentences make this difficult balance manifest: *Specific to network analysis, differential SPNs have the additional advantage of alleviating the concerns with the choice of a threshold value through the selection of a specific p-value. In essence, network thresholding is here supplanted by inference.*

5. P. 5, paragraph two. Again the discussion of advantages is easier to understand if it follows the authors presentation of the method.

The reviewer is correct to suggest that such discussion of the respective advantages of the mean and differential SPNs should be included later on in the manuscript. Thus, we have simplified this paragraph, as follows: *The problem of comparing networks over different experimental conditions centers on two distinct questions: (i) what are the nodes and (ii) what are the edges that significantly vary due to the experimental design. Statistical inference for these two distinct but related questions permit the construction of SPNs. One of the key advantages of this method is that it answers the problem posed by the quasi-linearity of the thresholding function highlighted in equation (1). Since we are drawing inference from the correlation coefficients themselves, we bypass the problem of averaging over a set of networks, while still providing a statistical summary in the form of a graph. In effect, we are trading the choice of a thresholding cut-off point with the use of a specific significance level.*

6. P. 5, last paragraph: Describe the data in terms of individual and condition specific correlation matrices arranged as matrix-valued elements in an nxJ array.

Yes, we agree that this is the content of that paragraph. Observe that the matrix $\mathbf{R}$ below is indeed an $n \times J$ array of matrices. Some small typos have also been corrected in that description in the revised paper: *The raw data of interest is an $(n \times J)$-matrix of correlation matrices, where the indexes $i = 1, \ldots, n$ and $j = 1, \ldots, J$ label the individual subjects and the conditions of the experiment of interest, respectively. The entire data set can thus be represented as the following matrix,*

$$\mathbf{R} = \begin{vmatrix} \mathbf{R}_{11} & \ldots & \mathbf{R}_{1J} \\ \vdots & \ddots & \vdots \\ \mathbf{R}_{n1} & \ldots & \mathbf{R}_{nJ} \end{vmatrix}, \tag{1}$$

where each element $\mathbf{R}_{ij}$ in equation (1) is a correlation matrix of order $N_V \times N_V$. These vertices are labelled by $v = 1, \ldots, N_V$. For simplicity, an entry in $\mathbf{R}$, will be denoted by r^e_{ij}, where $e \in E$ denotes an edge from the saturated graph G_S, which has the maximal number of edges –that is, $|E(G_S)| = N_V(N_V - 1)/2$. We will systematically use superscripts to denote edges.

7. P. 6: Equation (3) is not a vector. The equation clarifies its definition, but again, I suggest cleaning up the description similar to #6.

Following the comments of the first reviewer, this section has been discarded in the revised paper.

8. P. 6: Following the statement regarding the Fisher z-transformation, it would be beneficial to state that henceforth, the authors assume that rs have been suitably transformed. Subsequent discussion seems to presume use of correlation matrices.

We have added a sentence to that effect in the revised paper: *In the sequel, it will be assumed that all the correlation coefficients under discussion have been suitably Fisher-transformed.*

9. P. 7, equation (8): Discuss assumptions for b^e_i and ϵ^e_i. What covariance structures are applicable in context of fMRI network studies? Describe challenges that one may face when applying the model. For example, if one includes random intercepts (b_{i0}) and slope (b_{i1}) terms over increasing memory demand, it is likely that $b_{i1} \approx 0$ for some edges, which may require large samples for estimation given that the parameter is near the boundary of the parameter space. Also, computation time may become unwieldy when a graph contains a large number of edges and depending on the parametric structure assumed for the variance-covariance matrix $Var(b^e_i, \epsilon^e_i)$. Except for special settings, estimation requires the use of iterative algorithms. How does one check for convergence among the many edges? These and other relevant

considerations should be discussed.

This mixed effects model is applied independently elementwise for each edge. Therefore, the computational cost for fitting each model is relatively small. A computational bottleneck may be encountered if we deal with a very large number of edges. However, this grows only quadratically with the number of vertices. The independence assumption, however, is not tenable, and we therefore need to control for multiple comparisons. As for most applications of mixed effects models, we have assumed that the covariance matrix for the error terms, β is diagonal, whereas the one for the random effects is a full covariance matrix, simply assumed to be positive semi-definite. We have added a small sentence in the revised manuscript, which reflects these assumptions: *As in standard applications of mixed effects models, the covariance matrices for* $\boldsymbol{\epsilon}^e$ *and* $\mathbf{b}^e$ *were assumed to be diagonal and positive semi-definite, respectively (see Demidenko, 2004, for details).*

10. The authors should discuss a similar modeling framework proposed by Simpson et al. (2013), http://arxiv.org/ftp/arxiv/papers/1302/1302.5721.pdf (see equation 14).

We thank the reviewer for mentioning this interesting paper, and for highlighting some recent developments in the literature. We have added the following paragraph at the end of section 2.2, in order to report these new advances. *Note that the Frechet framework discussed in this section is substantially different from the use of exponential random graph models (ERGMs) (see Kolaczyk, 2009, for a review). Such models have been extended to include temporal variants, which are particularly suitable for the analysis of functional MRI data (Snijders et al., 2010b,a). When using ERGMs, however, one directly models the random process underlying the generation of the observed graphs. The Frechet framework, by contrast, solely relies on the definition of a metric between every pair of graphs, and therefore makes fewer mathematical assumptions.*

11. I would remove mention of growth curve models unless the authors provide more clarity around the specific form of the proposed model. Growth curves may be fit as a special case of the linear mixed model in (8). Also, there is a much longer (and older) literature on classic growth curve models for multivariate linear models (Grizzle and Allen, 1969; Rao, 1973; and many others).

The reviewer is right to point out that such details are irrelevant and potentially misleading. We have therefore suppressed every mention of the phrase "growth curve model", and have simplified this part of the manuscript.

12. P. 8: In the sentence following equation (9), the superscript e should be v.

This has been modified, as suggested.

13. P. 8: The authors state a node was judged to be either significantly upweighted or significantly downweighted depending on the sign of the largest coefficient in the corresponding vector β^v. The rationale is not completely clear to this reviewer, for example, if a study involves both an inhibitory and excitatory task.

Indeed, this type of labeling of the different coefficients, based on the sign of the beta's is only applicable, when one considers a task, based on an experimental gradient, such as the N-back task. We have added one sentence that describes this limitation: *Naturally, this assignment based on the sign of the* β^v*'s is only possible, when the task under scrutiny is based on an experimental gradient. An alternative strategy may be required, when different levels of the task are expected to affect the response in different directions.*

14. P. 8: Mixed models for voxel or region level summary statistics (such as equation 9) have been proposed by several authors for the analysis of fMRI data. The authors should make reference to some of these, e.g. Bowman (JASA, 2007) and Kang (JASA, 2012).

We thank the reviewer for pointing this out. We have added a reference to Bowman (2007), which reflects the work of these authors: *For both edge- and node-specific differential SPNs, several authors such as Bowman (2007) have made extensive use of mixed effects models (see Demidenko, 2004, for a comprehensive treatment).*

15. P. 9: The authors propose the use of a restricted Frechet mean estimator. This estimator has nice large-sample properties, with respect to the theoretical restricted Frechet mean. I envisage that as $n_j \to \infty$, some optimal large-sample properties also hold with respect to the true Frechet mean under the classical definition. In context of neuroimaging studies, which typically involve small sample sizes, it would be beneficial for the authors to discuss what is known about the performance of the restricted Frechet mean estimator in small samples or discuss potential limitations in performance.

Indeed, the speed of convergence is proportional to the sample size. We have added the following sentence to indicate this limitation: *However, since neuroimaging data sets tend to be characterized by small sample sizes, one should nonetheless exert caution, when applying these methods in this context.*

16. P. 12: K is assumed to follow a discrete uniform distribution. Would one expect less probability mass at the extremes, e.g. at 1?

We have considered a more variegated range of distribution on K in a previous paper, which has been referenced extensively in this manuscript. We have added the following sentence to refer the readers to this work: *Note, however, that other distributions could be selected for this purpose (see Ginestet et al., 2011, for a discussion of alternative specifications).*

Other editorial suggestions:

a. The second sentence of the abstract seems redundant. I suggest deleting the second sentence and changing the first sentence to something along the lines of Comparing either weighted networks or unweighted networks is hard because the topological properties

This is entirely correct. We have modified the first two sentences by dropping any mention to weighted networks in the first sentence, in order to ensure that both sentences convey independent pieces of information: *Comparing networks in neuroscience is hard, because the topological properties of a given network are necessarily dependent on the number of edges in that network. This problem arises in the analysis of both weighted and unweighted networks.*

b. P. 3, first sentence of section 2: change wording to describe how one can construct

This has been modified, as suggested.

c. P. 4, first sentence: change wording to Here, we review

This has been modified, as suggested.

d. P. 4, paragraph 1: change MRI to fMRI in SPM revolutionized the analysis of PET and later MRI data

This sentence has been deleted from the revised manuscript.

e. In the same sentence as (d) above, change regions of interest. ROIs are typically designated on the front end of an analysis rather than being interpreted as the spatial locations revealed from an analysis, e.g. reflecting locations of task-related changes in neural activity. I suggest changing regions of interest to something like distributed patterns of task-related changes in activity or metabolism

This sentence has been deleted from the revised manuscript.

f. P. 4, second sentence of paragraph two: latter should be former. g. P. 4. Change entrywise to elementwise.

We believe that our use of 'latter' was actually appropriate, in that sentence. However, we have replaced entrywise by elementwise, as suggested by the reviewer.

h. P. 6, last paragraph: change Inference testing to Hypothesis testing.

This sentence has been deleted from the revised manuscript.

i. P. 14: change shown that modularity structure substantially mediated by to shown that modularity structure is substantially mediated by.

This has been modified, as suggested.

References

Achard, S. and Bullmore, E. (2007). Efficiency and cost of economical brain functional networks. *PLOS Computational Biology*, **3**, 174–182.

Achard, S., Salvador, R., Whitcher, B., Suckling, J., and Bullmore, E. (2006). A resilient, low-frequency, small-world human brain functional network with highly connected association cortical hubs. *J. Neurosci.*, **26**(1), 63–72.

Bassett, D.S., Bullmore, E., Verchinski, B.A., Mattay, V.S., Weinberger, D.R., and Meyer-Lindenberg, A. (2008). Hierarchical organization of human cortical networks in health and schizophrenia. *J. Neurosci.*, **28**(37), 9239–9248.

Benjamini, Y. and Hochberg, Y. (1995). Controlling the false discovery rate: A practical and powerful approach to multiple testing. *Journal of the Royal Statistical Society. Series B (Methodological)*, **57**(1), 289–300.

Bollobás, B. (1998). *Modern Graph Theory.* Springer, London.

Bowman, F.D. (2007). Spatiotemporal models for region of interest analyses of functional neuroimaging data. *Journal of the American Statistical Association*, **102**(478), 442–453.

Demidenko, E. (2004). *Mixed Models: Theory and Applications.* Wiley, London.

Friston, K.J. (1994). Functional and effective connectivity in neuroimaging: A synthesis. *Human Brain Mapping*, **2**(1-2), 56–78.

Ginestet, C.E., Best, N.G., and Richardson, S. (2012). Classification loss function for parameter ensembles in bayesian hierarchical models. *Statistics and Probability Letters*, **82**(0), 859–863.

Ginestet, C.E., Nichols, T.E., Bullmore, E.T., and Simmons, A. (2011). Brain network analysis: Separating cost from topology using cost-integration. *PLoS ONE*, **6(7)**, e21570.

Ginestet, C.E. and Simmons, A. (2011). Statistical parametric network analysis of functional connectivity dynamics during a working memory task. *NeuroImage*, **5(2)**, 688–704.

He, Y., Chen, Z.J., and Evans, A.C. (2007). Small-world anatomical networks in the human brain revealed by cortical thickness from MRI. *Cereb. Cortex*, **17**(10), 2407–2419.

He, Y., Wang, J., Wang, L., Chen, Z.J., Yan, C., Yang, H., Tang, H., Zhu, C., Gong, Q., Zang, Y., and Evans, A.C. (2009). Uncovering intrinsic modular organization of spontaneous brain activity in humans. *PLoS ONE*, **4**(4), 1–18.

Kolaczyk, E. (2009). *Statistical Analysis of Network Data: Methods and Models*. Springer-Verlag, London.

Meskaldji, D.E., Ottet, M.C., Cammoun, L., Hagmann, P., Meuli, R., Eliez, S., Thiran, J.P., and Morgenthaler, S. (2011). Adaptive strategy for the statistical analysis of connectomes. *PloS one*, **6**(8), e23009–.

Nichols, T. and Hayasaka, S. (2003). Controlling the familywise error rate in functional neuroimaging: a comparative review. *Statistical Methods in Medical Research*, 419–446.

Snijders, T.A., Koskinen, J., and Schweinberger, M. (2010a). Maximum likelihood estimation for social network dynamics. *The Annals of Applied Statistics*, **4**(2), 567–588.

Snijders, T.A., Van de Bunt, G.G., and Steglich, C.E. (2010b). Introduction to stochastic actor-based models for network dynamics. *Social networks*, **32**(1), 44–60.

van Wijk, B.C.M., Stam, C.J., and Daffertshofer, A. (2010). Comparing brain networks of different size and connectivity density using graph theory. *PLoS ONE*, **5**(10), 13701–13716.
